# Do intentions lead to action? Results of a longitudinal study assessing determinants of Tdap vaccine uptake during pregnancy in Quebec, Canada

**DOI:** 10.1186/s12884-022-04809-6

**Published:** 2022-06-13

**Authors:** Eve Dubé, Marie-Eve Trottier, Maryline Vivion, Manale Ouakki, Nicholas Brousseau, Maryse Guay, Zineb Laghdir, Isabelle Boucoiran, Bruce Tapiéro, Caroline Quach

**Affiliations:** 1 Direction des risques biologiques et de la santé au travail, Institut national de la santé publique du Québec, Eve Dubé, 2400 D’Estimauville, Québec, QC G1E 7G9 Canada; 2 Axe maladies infectieuses et immunitaires, Centre de recherche du CHU de Québec -Université Laval, Quebec City, Qc Canada; 3 Direction de la valorisation scientifique et qualité, Institut national de la santé publique du Québec, Québec City, Qc Canada; 4 Département des sciences de la santé communautaire, Faculté de médecine et des sciences de la santé, Université de Sherbrooke, Sherbrooke, Qc Canada; 5 CHU Sainte-Justine Research Center, Montreal, Qc Canada; 6 Department of Obstetrics & Gynecology and School of Public Health, Université de Montréal, Montréal, Qc Canada; 7 Infectious Diseases Division, Department of Pediatrics, CHU Sainte-Justine, Montreal, Qc Canada; 8 Departments of Microbiology, Infectious Diseases and Immunology and of Pediatrics, Université de Montréal, Montreal, Qc Canada; 9 Infection Prevention and Control, Department of Clinical Laboratory Medicine, CHU Sainte-Justine, Montreal, Qc Canada

**Keywords:** Immunization, Pertussis, Pregnancy, Public health, Knowledge, attitudes

## Abstract

**Background:**

In Canada, vaccination against pertussis (Tdap) during pregnancy has been recommended since 2018, with suboptimal uptake. We aimed to assess the determinants of intention and uptake of Tdap vaccine among pregnant women in Quebec.

**Methods:**

Participants (< 21 weeks of pregnancy) were recruited in four Quebec regions. Two online surveys were administered during pregnancy (< 21 weeks and > 35 weeks). One measured vaccination intention and the other assessed the actual decision. Questionnaires were informed by the Theory of Planned Behaviour (TPB). We used logistic multivariate analysis to identify determinants of Tdap vaccination uptake during pregnancy using responses to both questionnaires.

**Results:**

A total of 741 women answered the first survey and 568 (76.7%), the second survey. In the first survey most participants intended to receive the Tdap vaccine during their pregnancy (76.3%) and in the second survey, 82.4% reported having been vaccinated against Tdap during their pregnancy. In multivariate analysis, the main determinants of vaccine uptake were: a recommendation from a healthcare provider (OR = 7.6), vaccine intention (OR = 6.12), social norms (or thinking that most pregnant women will be vaccinated (OR = 3.81), recruitment site (OR = 3.61 for General Family Medicine unit) perceived behavioral control (or low perceived barriers to access vaccination services, (OR = 2.32) and anticipated feeling of guilt if not vaccinated (OR = 2.13). Safety concerns were the main reason for not intending or not receiving the vaccine during pregnancy.

**Conclusion:**

We observed high vaccine acceptance and uptake of pertussis vaccine in pregnancy. The core components of the TPB (intention, social norms and perceived behavioral control) were all predictors of vaccine uptake, but our multivariate analysis also showed that other determinants were influential: being sufficiently informed about Tdap vaccination, not having vaccine safety concerns, and anticipated regret if unvaccinated. To ensure high vaccine acceptance and uptake in pregnancy, strong recommendations by trusted healthcare providers and ease of access to vaccination services remain instrumental.

**Supplementary Information:**

The online version contains supplementary material available at 10.1186/s12884-022-04809-6.

## Background

In addition to social and economic disruptions, the pandemic of COVID-19 impacted routine vaccination programs in Canada. School-based programs were halted due to school closures, but prenatal and infant vaccination were continued as this health sector was prioritized at the beginning of the pandemic [1]. Vaccination has become part of the standard routine care during pregnancy [2]. Vaccination against pertussis during pregnancy has been shown to protect the infant through its first 3 months of life [3–5]. Since May 2018, the tetanus-diphtheria-acellular pertussis (Tdap) vaccine has been recommended to women, during every pregnancy, in Canada and Quebec [3, 6]. However, vaccination coverage among pregnant women remains suboptimal. The component on Vaccination During Pregnancy of the Childhood National Immunization Coverage Survey (CNICS) showed that only 43 and 49% of pregnant women received the Tdap vaccine between March 2018 and March 2019 in Canada and in the province of Quebec, respectively [7]. The main reason for not being vaccinated was the lack of knowledge that the vaccine was recommended during pregnancy [7]. A qualitative study conducted among pregnant women in Quebec prior to the program onset also showed a lack of awareness regarding vaccination in pregnancy and important safety concerns among participants otherwise favorable to vaccination [8]. Many studies have assessed determinants of vaccination during pregnancy and have shown that barriers to vaccine uptake are diverse, and include a lack of awareness, lack of acceptance, or lack of access to vaccination services [9]. Other reasons such as risk perception of vaccines during pregnancy could also influence women’s decision for the Tdap vaccine [10]. Furthermore, the negative influence of misinformation about vaccines is often invoked as a cause of lower vaccine acceptance and uptake among parents and pregnant people [11]. The aim of this longitudinal questionnaire-based study was to assess the determinants of intention and uptake of Tdap vaccine among pregnant women in Quebec.

## Methods

Longitudinal surveys were conducted as part of a larger quasi-experimental multicenter study with non-equivalent control groups of pregnant women, that took place in 4 health care regions of Quebec, Canada [12].

### Recruitment of participants

We used a quasi-experimental multicenter study design with non-equivalent control groups of pregnant women in 4 different regions in the province of Québec, Canada (Montréal, Montérégie, Capitale-Nationale and Mauricie) [12]. These settings were selected because they represent a variety of ways for pertussis vaccine delivery to pregnant women (i.e. Local community Services Center (CLSC), Family Medicine Group (FMG), Vaccination offered during the Oral Glucose Challenge Test (OGCT) in an academic hospital). From April to October 2019, women < 21 weeks of pregnancy were recruited during a routine visit with their regular health care provider or during their appointment for blood work. Eligible participants were 18 years of age and over, spoke French or English, and provided a valid email address.

### Data collection

Participants answered a recruitment questionnaire (< 21 weeks of pregnancy) and two different online surveys (second trimester + third trimester). Contact information and sociodemographic characteristics (age, place of birth, number of pregnancies, etc.) were collected at recruitment by the research staff. Women were invited to fill a first online questionnaire during their second trimester of pregnancy and a second during their third trimester. We also asked permission to access the vaccination registry or medical charts for more information on vaccine uptake. For each online questionnaire, a total of three email reminders were sent, if necessary.

The questionnaires’ development was informed by Ajzen’s Theory of Planned Behaviour (TPB). TPB implies that behaviours are predicted by personal intentions, if the environment is conductive [13, 14]. According to the TPB, the intention is based on three core components: attitudes toward the behaviour (i.e., the degree to which an individual has a favorable or unfavorable evaluation of the behavior), perceived behavioral control (i.e., individual’s perceived ease or difficulties in performing the behaviour) and social norms (i.e., social pressures and what an individual think significant other would think of the behaviour).

The second trimester questionnaire assessed vaccination intention for Tdap during pregnancy and included items derived from the TPB and other items such as perceived risk of pertussis for infants, past vaccination behaviour and anticipated regret (i.e., anticipated feeling of sorrow an individual anticipates if a negative event occurs if not vaccinated) (see Appendix 1 for the full list of survey items). Attitudes’ items measured participants’ perceptions of vaccine safety and efficacy, perceived vaccine usefulness and perceived risks of pertussis for themselves and their babies. For norms, items measured personal normative beliefs (what significant others would want the participant to do regarding vaccination in pregnancy) and subjective norms (what the participants thinks other pregnant women are doing about vaccination). Questions about direct and indirect perceived behavioral control questions were also added. The third trimester questionnaire measured vaccination decision (self-reported vaccine receipt) and reasons behind vaccination decision (e.g., recommendations by a healthcare provider, opportunity to be vaccinated during a routine appointment, etc.). To ensure ease of understanding, all questions were in relation with “vaccination against pertussis in pregnancy” and the vaccine formulation (Tdap) was not used.

Most items in both questionnaires were closed-ended questions with responses formatted on a six-point scale ranging from “Strongly disagree” to “Strongly agree” (Appendix 1). The survey was pretested, and minor adjustments were made in the wording of some questions. Only two questions were mandatory (i.e., vaccine intention in the first questionnaire and vaccine uptake in the second questionnaire), but otherwise participants could skip questions.

### Data analysis

Descriptive statistics were generated for all items of both questionnaires. Vaccine uptake responses were validated in the Quebec’s Immunization Registry and participants for whom the third trimester’s survey responses were discordant with data in the registry or who answered “I don’t know” were excluded. As of January 2019, all vaccines administered to Quebec residents must be recorded in the registry [15]. Participants whose vaccination status that was not found in the registry or in medical charts, who experienced miscarriage or premature birth, or who moved out of the province of Quebec were excluded.

Each sub construct belonging to the TPB was measured with three different items, except for perceived direct behavioral control and personal normative beliefs who each had two items. The internal consistency of multiple-item theoretical constructs was calculated with the Cronbach’s alpha coefficient of > 0.7. The mean of all items belonging to the same construct was also calculated to have a global measure of the construct. Logistic multivariate analysis was used to determine variables independently associated with the participants’ intention to take the Tdap vaccine and the vaccine uptake during pregnancy. A combination of hierarchical and stepwise procedures was used to determine which variables, potentially associated with probability of vaccination, would be kept in the final model (*p* < 0.05). Individual items (i.e. sociodemographic, other constructs than TPB) or global construct scores (for theoretical construct) associated with intention and vaccine uptake at *p* < 0.10 in univariate analysis were included in the logistic regression models and kept in the final models with *p* < 0.05. The collinearity was checked, and the model fit was assessed by the Hosmer and Lemeshow test. Statistical significance was based on *p* < 0.05 (2-sided). Variables with missing values were excluded from the adjusted analysis. Both models were adjusted for the vaccination sites because it was potentially a confounding variable. Statistical analysis was conducted using SAS, version 9.4 (SAS Institute, Cary, NC). Bivariate analyses were based on Chi-2 test or Fisher exact test as appropriate.

## Results

**Fig. 1:**
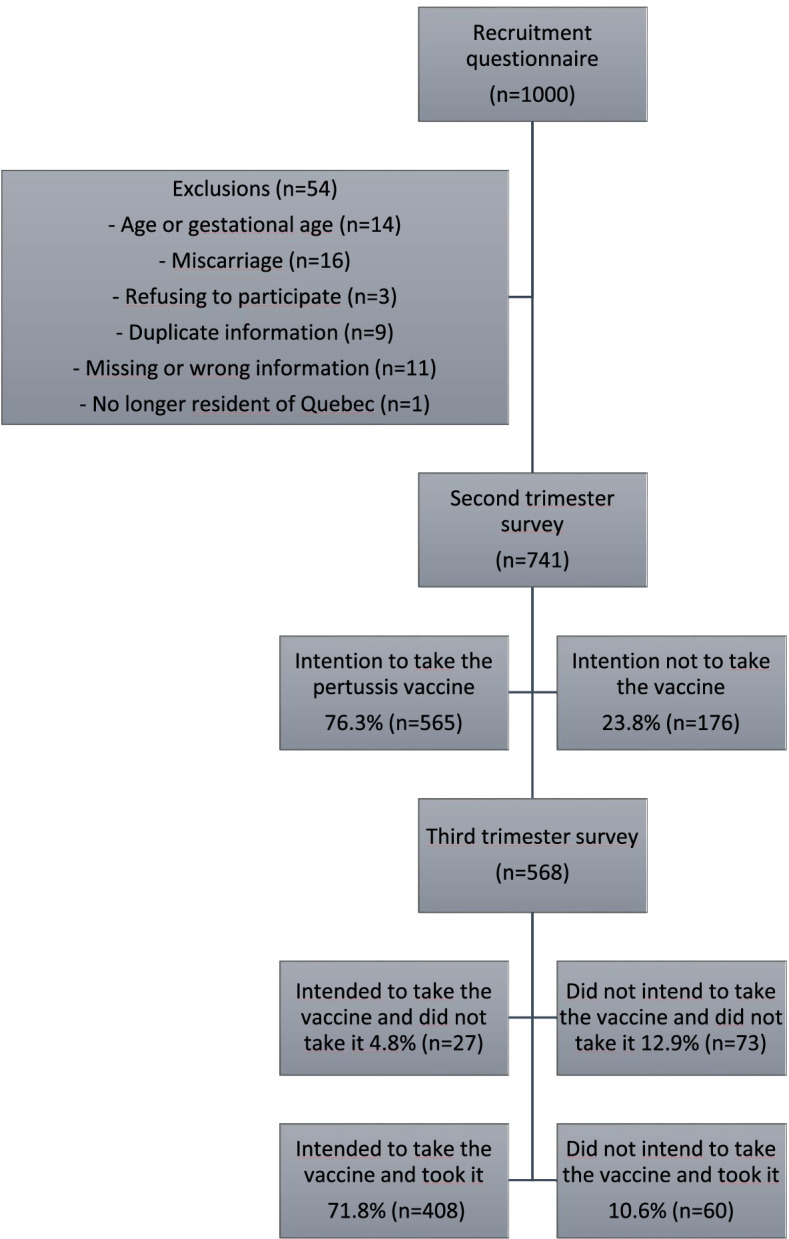
Decisional flowchart

In total, 1000 pregnant women were recruited, and 946 women were eligible to participate after the exclusion of 54 women (14 based on age or gestational age, 16 who miscarried, 3 who later refused to participate, 9 with duplicate information, 11 with missing or wrong information, and 1 no longer residing in Quebec) Fig. 1.

Of these 946 women, 741 answered the second trimester survey and 568 of them, the third trimester survey. Table 1 presents participants’ characteristics according to vaccination uptake. Most participants were ≥ 30 years of age (60.9%), born in Canada (74.7%), French speaking (82.8%), married (91.6%), and had a university degree (60.6%). Almost half (48.1%) were in their first pregnancy (Table 1).

**Table 1:** Sociodemographic characteristics of participants according to self-reported Tdap vaccination during pregnancy^a^

	Vaccine status				Total
	Vaccinated		Not vaccinated		
**Characteristics of participants**	**n**	**%**	**n**	**%**	**n (%)**
**Total**	**468**	**82.4**	**100**	**17.6**	**100.0**
**Age**					
< 30 years old	184	39.3	38	38.0	39.1
≥ 30 years old	284	60.7	62	62.0	60.9
Country of birth**					
Canada	364	77.8	60	60.0	74.7
Outside Canada	90	19.2	35	35.0	22.0
Unknown	14	3.0	5.0	5.0	3.4
First language**					
French	399	85.3	71	71.0	82.8
English	17	3.6	2	2.0	3.4
Other	52	11.1	27	27.0	13.9
**Level of education**					
High school or less	83	17.7	25	25.0	19.0
College	99	21.2	16	16.0	20.3
University	285	60.9	59	59.0	60.6
Other	1.0	0.2	0.0	0.0	0.2
**Marital status**					
Prefer not to answer	3	0.6	0.0	0.0	0.5
Single/Divorced	36	7.7	8	8.0	7.8
Married / Common-law partner	429	91.7	91	91.0	91.6
Other	0.0	0.0	1.0	1.0	0.2
**N of pregnancies**					
1	231	49.4	43	43.0	48.2
2	166	35.5	36	36.0	35.6
≥ 3	71	15.2	21	21.0	16.2
**Health care professional following pregnancy**					
N/A	6	1.3	0	0.0	1.1
Family physician or general practitioner	143	30.6	23	23.0	29.2
Gynecologist/ Obstetrician	298	63.7	73	73.0	65.3
Midwife	0.0	0.0	1	1.0	0.2
Other	21	1.5	3	3.0	1.4
**Vaccination sites**					
University Hospital	125	26.7	54	54.0	31.5
Gynecology and Obstetrics Clinic	127	27.1	7	7.0	23.6
Family medicine group	96	20.5	12	12.0	19.0
Local community service centre	120	25.6	27	27.0	25.9

^a^Missing values were excluded, this explains when % do not add up to 100%

**P value calculated without missing values. *P* value < 0.004

Overall, 82.4% of participants were vaccinated against Tdap during pregnancy. Statistically significant differences were found in vaccine uptake according to country of birth (*p* = 0.004), first language (*p* = 0.004) and vaccination sites (*P* < 0.0001).

### Determinants of participants’ intention to receive the Tdap vaccine during pregnancy

The internal reliability of the four components of the theoretical model was good. Cronbach’s alpha was between 0.62 and 0.87, except for direct perception of control (0.57). In the second trimester, 76.3% of mothers had positive intentions regarding Tdap vaccination during pregnancy. Vaccine intention was correlated with positive attitude toward vaccine efficacy (α = 0.70), vaccine safety (α = 0.87), and vaccination in general (α = 0.87). Indirect perceived behavioral control (α = 0.83) and subjective norms (α = 0.80) were also correlated with the intention of taking the vaccine.

Results of the multivariate analysis for the second trimester survey are shown in Appendix 2. Subjective norms score, positive attitudes toward Tdap vaccine, perception of having enough information to make a decision on Tdap vaccination during pregnancy, low fear of adverse events, anticipated regret (or anticipated feeling of guilt if not taking the Tdap vaccine), easy access to Tdap vaccine during pregnancy and positive attitudes toward effectiveness of the vaccine were factors found to influence the intention to take the vaccine.

### Determinants of participants’ receipt of the Tdap vaccine during pregnancy

Of the 76.3% women who intended to be vaccinated against pertussis, 93.8% acted on their intention and received the vaccine. Of the 23.4% of women who did not intend to get the vaccine, 45.1% were vaccinated. Among women who did not intend to get the vaccine but took it, the main reasons for it were: having received a recommendation from a health care provider (86.7%), having had the opportunity to be vaccinated during a routine appointment (41.7%), and perception that Tdap vaccination is useful to protect their babies health (56.7%) (Table 2). Similar reasons were mentioned by women who intended to be vaccinated in the second trimester and took the vaccine.

**Table 2:** Main reasons for being vaccinated against Tdap during pregnancy by intention^a^

	Intention to get the Tdap vaccine during pregnancy				Total (%)
	Yes		No		100%
	***n*** = 408	%	***n*** = 60	%	
**Having received a recommendation from a health care provider**	341	83.6	52	86.7	84.0
**Views that vaccine against Tdap is useful to protect my baby’s health**^**a**^	305	74.8	34	56.7	72.4
**Having had the opportunity to be vaccinated during a routine appointment**	175	42.9	25	41.7	42.7
**Ease to make an appointment to take the Tdap vaccine during pregnancy**^**a**^	127	31.1	10	16.7	29.3
Having received a recommendation from someone in my network**	23	5.6	10	16.7	7.1
**Other reasons**	13	3.2	4	6.7	3.6

^a^Participants could select more than one item

**p < 0.05

Out of 76.3% women who had the intention of being vaccinated in the second trimester questionnaire, 6.2% did not receive the vaccine and of the 23.4% who had no intention of being vaccinated, 54.9% did not receive it either. The main reasons for not being vaccinated among women who did not intend to do so were: fear of adverse events of the Tdap vaccine during pregnancy (32.9%), thinking that the Tdap vaccine during pregnancy was not important (23.3%) and thinking that the Tdap vaccine was not useful for their infant’s health (15.1%) (Table 3). For women who intended to take the vaccine but did not take it, the main reasons were: difficulty to make an appointment to be vaccinated during pregnancy (18.5%), it was complicated to have the vaccine (11.1%), and other reasons (59.3%).

**Table 3:** Univariate analysis: main reasons for not taking the Tdap vaccine during pregnancy by intentions

	Intention to take the Tdap vaccine during pregnancy				Total (%)
	Yes		No		Total
	***n*** = 27	%	***n*** = 73	%	100%
**Not knowing that a Tdap vaccine was given during pregnancy**	2	7.4	5	6.9	7.0
**Giving birth to a premature baby before having the chance to take the vaccine**	2	7.4	3	4.1	5.0
Thinking that the Tdap vaccine is not useful for the baby’s health*	0	0.0	11	15.1	11.0
Thinking it is not important to get the Tdap vaccine during pregnancy*	1	3.7	17	23.3	18.0
Fear of Tdap vaccination during pregnancy*	0	0.0	24	32.9	24.0
**Difficulty to make an appointment for the Tdap vaccine during pregnancy**	5	18.5	4	5.5	9.0
**Complicated to have the vaccine**	3	11.1	1	1.4	4.0
**My family, friends or spouse did not approve of me taking the vaccine**	0	0.0	7	9.6	7.0
Other reasons*	16	59.3	18	24.7	34.0

*p < 0.05

Most participants (95.5%) who received the vaccine had been informed by a health care provider about vaccination (85.5% by their own maternal care provider) (Appendix 3). In comparison, 63% of participants who were not vaccinated mentioned having been informed about Tdap vaccination in pregnancy (49% by their own maternal care provider). Written information about Tdap vaccination was given to 91.7% of women who were vaccinated compared to 69% of unvaccinated women. The main other sources of information reported by unvaccinated participants were: health care providers who don’t follow pregnancy (i.e. pharmacists, nurses or medical doctors’ consultations outside of the pregnancy context). For vaccinated participants, these other sources were: someone who promoted the study at their clinic, friend, health care provider that they talked to during the pregnancy.

Results of the multivariate analysis on determinants of Tdap vaccine uptake are shown in Table 4. Recommendations from a healthcare provider (OR = 7.6), intention to be vaccinated (OR = 6.12), subjective norms (OR = 3.81), vaccination sites (in a general family medicine unit (OR = 3.63) and in an obstetric clinic (OR = 2.97)), perceived behavioural control (e.g., ease of access to vaccination services) (OR = 2.32) and anticipated regrets (OR = 2.13) were significantly associated with Tdap vaccine uptake.

**Table 4:** Findings of multivariate analysis on determinants of the Tdap vaccine uptake

		OR	95%CI		*p* Value
Vaccination recommendation by a healthcare provider		7.26	3.25	16.26	<.0001
Intention to be vaccinated against Tdap during pregnancy		6.12	3.16	11.87	<.0001
Subjective norm (i.e., perceived support for vaccination by significant others)		3.81	1.90	7.61	0.0002
Recruitment site	FMG	3.61	1.47	8.86	0.0051
	Obstetric clinic	2.97	1.12	7.89	0.0288
Perceived behavioural control (i.e., perceived ease of being vaccinated in pregnancy)		2.32	1.23	4.37	0.0091
Anticipated regret (i.e., anticipated feeling of sorrow if something negative happens while unvaccinated)		2.13	1.13	4.00	0.0191

## Discussion

The longitudinal approach used in this study allowed us to identify determinants of Tdap vaccine acceptance and uptake in pregnancy, including perceived ease of access to vaccination services and impact of vaccine endorsement by health care providers. Our findings indicate a high level of maternal immunization acceptance and uptake, with 76.3% of participants intending to be vaccinated and 82.4% who, in the end, were vaccinated. This is above what has been observed in other studies conducted in Canada and could indicate that vaccination is becoming better integrated into routine pregnancy care [6, 8]. However, because our study was conducted prior to the pandemic, the impact of COVID-19 on routine immunization in pregnancy is still unknown.

In this study, congruent with the TPB that informed the development of our questionnaires, one of the main determinants of pertussis vaccine uptake was the intention to be vaccinated, as we also see in other studies about acceptance of other vaccines in general [16]. However, our findings bring a new dimension to the field of research on vaccine acceptance in pregnancy: it is possible to modify negative intentions and overcome initial reluctance to vaccinate. Of the 23.3% of participants who had no intention of being vaccinated, 45.1% finally were. Receiving a recommendation from a maternal care provider, the opportunity to be vaccinated during a routine care visit and reassurance about Tdap vaccine safety and usefulness were key influencers in these participants’ decision to finally accept the vaccine. In contrast, 6.6% of participants who intended to be vaccinated were not vaccinated. The main barriers faced by these participants were related to access issues, fear of adverse events for themselves or their fetus, and low perceived usefulness of the vaccine. Barriers to vaccination in pregnancy identified in this study are congruent with the literature [9, 17]. However, findings highlight that interventions to address vaccine hesitancy in pregnancy should be coupled with easy access to vaccination for optimal success.

Vaccination during pregnancy is a single intervention that has the potential to protect newborns against pertussis and its complications. Logistical issues in delivering vaccines to pregnant women along with lack of knowledge or negative beliefs in both maternal care providers and pregnant women have been identified as significant barriers to vaccine uptake in pregnancy in our study and others [9, 18]. Our study showed that receiving a vaccination recommendation from a trusted health care provider is a key factor in enhancing vaccine uptake in pregnancy; the importance of providers’ recommendations is well-recognized in the literature [19–26]. Unfortunately, studies have shown that many maternal care providers consider not having enough time during their consultation to inform and educate patients about vaccination during pregnancy or do not view vaccine administration as part of their scope of practice [26–28].

TPB components also predicted vaccine uptake and our multivariate analysis also showed that other determinants were influential to vaccine uptake: being sufficiently informed about Tdap vaccination, low perceived vaccine safety concerns, and anticipated regret. These determinants are known to influence vaccination decisions in general [29] and during pregnancy in particular [30].

Our study has some limitations. First, 50% of participants had a university degree which is high compared to the general population. A selection bias is possible as most participants were recruited in healthcare settings where providers have positive views and are proactive about maternal immunization. If other recruitment sites were added where health care providers had different views about vaccines and approach to pregnancy, this could have impacted vaccine acceptance and uptake [31–34]. Although we used TPB to inform the development of our questionnaires, the internal consistency of some constructs was low and below the threshold considered acceptable. We thus have included individual items in our multivariate analysis. Recall bias could potentially have influenced the findings, but should be minimal as the questionnaires were sent closely after the moment when vaccines should have been administered. Finally, a desirability bias is possible (i.e., participants provide responses that they think the researchers would want to see). However, the vaccine uptake was validated in the Quebec Immunization Registry. It is also possible that participation in this study had a positive influence on pregnant women’s attitudes and vaccination decisions because they were aware that the Tdap vaccine was given during pregnancy. Our data were collected prior to the pandemic and it is possible that pregnant persons were concerned about the safety of going to medical appointments [35] aiming at decrease pertussis vaccine uptake after. After we collected our data.

## Conclusions

In conclusion, this study provides valuable insight on knowledge, attitudes and behaviours about pertussis vaccination during pregnancy. Although it is possible that participants in a vaccine-related study have more favourable attitudes toward vaccination, our study’s findings indicate that it is possible to overcome initial vaccine hesitancy and enhance vaccine acceptance and uptake in pregnancy. It is important to ensure easy access to vaccination services within routine pregnancy care and vaccine recommendation by health care providers to increase vaccine acceptance and uptake in pregnancy. Communication strategies should reinforce vaccine safety and usefulness in pregnancy.

## Supplementary Information

**Additional file 1: Appendix 1.** Items in recruitment questionnaire.

**Additional file 2: Appendix 2.** Results of multivariate analysis on determinants of vaccine intention.

**Additional file 3: Appendix 3.** Table 6. Univariate analysis: information sources about Tdap vaccination during pregnancy and vaccination status*.

## Data Availability

The datasets used and/or analysed during the current study available from the corresponding author on reasonable request.
