# Supplementary material for: Do intentions lead to action? Results of a longitudinal study assessing determinants of Tdap vaccine uptake during pregnancy in Quebec, Canada
Source: BMC Pregnancy Childbirth. 2022 Jun 13;22:477. doi: 10.1186/s12884-022-04809-6 (PMC9189261; doi:10.1186/s12884-022-04809-6)
Supplement: Supplementary file 1 — Additional file 1: Appendix 1. Items in recruitment questionnaire. [file 12884_2022_4809_MOESM1_ESM.docx]

**Appendix 1**

**Items in recruitment questionnaire**

| **Question** | **Answer** | **TBP or theme** |
| --- | --- | --- |
| Age | 4 choices | Sociodemo |
| Are you born in Canada | Yes/No | Sociodemo |
| If no, what year you arrived in Canada | Open-ended. | Sociodemo |
| What is your matrimonial status | 6 choices | Sociodemo |
| Education | 5 choices including open-ended question (other) | Sociodemo |
| Language | 4 choices including open-ended question (other) | Sociodemo |
| How many children do you have? | Open ended + choice (this is my first pregnancy) | Sociodemo |
| Do you have diabetes? | 3 choices | Sociodemo |
| Who is the healthcare provider that follows your pregnancy? | 4 choices including open-ended question (other) | Sociodemo |
| Do you take part in programs during your pregnancy? | 3 choices including open-ended (other) | Sociodemo |
| When is your due date? | Open-ended | Sociodemo |

**Items in the second trimester questionnaire**

|  | **Items** | **TBP constructs or themes (scale of 6 choices going from “totally agree to totally disagree)** |
| --- | --- | --- |
| 1 | I plan of taking the pertussis vaccine during pregnancy | Intention |
| 2 | My personal values will incite me to take the pertussis vaccine during pregnancy | Perceived behavioral control |
| 3 | I am at risk to get pertussis | Attitudes |
| 4 | My baby is a risk to get pertussis | Attitudes |
| 5 | If I get pertussis, it will have severe consequences on my baby | Attitudes |
| 6 | I am worried about the possibility that my baby gets pertussis | Attitudes |
| 7 | Pertussis is a severe disease for a baby | Attitudes |
| 8 | Taking the pertussis vaccine during pregnancy will protect my baby during his first 3 months of life. | Attitudes |
| 9 | Taking the vaccine against pertussis during pregnancy will protect me | Attitudes |
| 10 | If I don’t take the vaccine against pertussis during pregnant, I will feel guilty | Attitudes |
| 11 | Fear of the vaccine’s adverse effects could discourage me to take it during pregnancy. | Attitudes |
| 12 | It is safe for a pregnant woman to take the pertussis vaccine. | Attitudes |
| 13 | Pertussis vaccine is effective for pregnant women | Attitudes |
| 14 | Vaccination against pertussis during pregnancy is safe for the fetus | Attitudes |
| 15 | If I breastfeed my baby, it is not necessary to take the pertussis vaccine during pregnancy | Attitudes |
| 16 | I am scared of vaccines in general | Attitudes |
| 17 | It is not safe to take the pertussis vaccine during pregnancy | Attitudes |
| 18 | If a health care provider tells me to take the vaccine, it will encourage me to take the vaccine during pregnancy | Norms |
| 19 | Difficulty to make an appointment to get the vaccine could discourage me from getting immunized during pregnancy | Perceived behavioral control |
| 20 | The fact that the vaccine is given thru a routine medical appointment during pregnancy will incite me to take the vaccine | Perceived behavioral control |
| 21 | Difficulty to access the clinic (i.e. hours or traffic) could discourage me from taking the vaccine during pregnancy | Perceived behavioral control |
| 22 | It will be easy for me to receive the pertussis vaccine during pregnancy | Perceived behavioral control |
| 23 | Most people close to me would give me the advice to take the pertussis vaccine during pregnancy | Norms |
| 24 | Most pregnant women around me took the vaccine against pertussis | Norms |
| 25 | Every pregnant woman should take the pertussis vaccine | Norms |
| 26 | I feel enough informed to make a decision about the pertussis vaccine during pregnancy | Perceived behavioral control |

**Items in the third trimester questionnaire**

| **Question** | **Answer** | **TBP or theme** |
| --- | --- | --- |
| Did you take the pertussis vaccine during your pregnancy? | Yes/no | Behavior |
| **Women who decided to take the vaccine** | | |
| When did you receive the vaccine during your pregnancy? | Open-ended | Factual information |
| Where did you take the vaccine? | 5 choices + Open-ended (other) | Factual information |
| Why did you take the pertussis vaccine during pregnancy? (Select all reasons that apply) | 5 choices + Open-ended (other) | Reasons for taking the vaccine |

|  | **Items** | **TBP constructs or themes TBP constructs or themes (scale of 6 choices going from “totally agree to totally disagree)** |
| --- | --- | --- |
| **1** | Other pregnant women around me took the pertussis vaccine | **Subjective norm** |
| **2** | I felt enough informed to make a decision on vaccination against pertussis during pregnancy | **Perceived behavioral control** |
| **3** | I felt comfortable to discuss about vaccination against pertussis during pregnancy with my healthcare provider | **Attitudes** |
| **4** | What I experienced during vaccination against pertussis during pregnancy was positive | **Attitudes** |
| **5** | If I had another pregnancy, I would not hesitate to take the vaccine | **Perceived behavioral control** |

| **Question** | **Answer** | **TBP or theme** |
| --- | --- | --- |
| Did a health care provider discuss vaccination against pertussis during pregnancy with you? | Yes/No/I don’t remember  Who: 3 choices Yes/No  Other: yes/no:  Open-ended | Information sources |
| Did you receive written information of vaccination against pertussis during pregnancy? | Yes/No/I don’t remember | Information sources |
| **For women who did not receive the vaccine** | | |
| What are your main reasons for not taking the vaccine? | 9 choices + including 3 open-ended precision answers | Reasons for the behavior |

|  | **Items** | **TBP constructs or themes TBP constructs or themes (scale of 6 choices going from “totally agree to totally disagree)** |
| --- | --- | --- |
| **1** | Other pregnant women around me took the pertussis vaccine | **Subjective norm** |
| **2** | I felt enough informed to make a decision on vaccination against pertussis during pregnancy | **Perceived behavioral control** |
| **3** | I felt comfortable to discuss about vaccination against pertussis during pregnancy with my healthcare provider | **Attitudes** |
| **4** | What I experienced during vaccination against pertussis during pregnancy was positive | **Attitudes** |
| **5** | If I had another pregnancy, I would not hesitate to take the vaccine | **Perceived behavioral control** |

| **Question** | **Answer** | **TBP or theme** |
| --- | --- | --- |
| Is there information linked to pertussis vaccination during pregnancy that you would like to share? | Open-ended |  |
| Did a health care provider discuss vaccination against pertussis during pregnancy with you? | Yes/No/I don’t remember  Who: 3 choices Yes/No  Other: yes/no:  Open-ended | Information sources |
| Did you receive written information of vaccination against pertussis during pregnancy? | Yes/No/I don’t remember | Information sources |
