# Supplementary material for: Do intentions lead to action? Results of a longitudinal study assessing determinants of Tdap vaccine uptake during pregnancy in Quebec, Canada
Source: BMC Pregnancy Childbirth. 2022 Jun 13;22:477. doi: 10.1186/s12884-022-04809-6 (PMC9189261; doi:10.1186/s12884-022-04809-6)
Supplement: Supplementary file 2 — Additional file 2: Appendix 2. Results of multivariate analysis on determinants of vaccine intention. [file 12884_2022_4809_MOESM2_ESM.docx]

**Appendix 2**

**Results of multivariate analysis on determinants of vaccine intention**

| Item | OR | 95%CI | | p value |
| --- | --- | --- | --- | --- |
| Subjective norms score (ref≥30) | 4.64 | 2.651 | 8.122 | <.0001 |
| Positive attitude toward Tdap vaccination (score) | 4.376 | 2.294 | 8.347 | <.0001 |
| Perception of having enough information to make a decision on Tdap vaccination during pregnancy | 3.462 | 2.024 | 592 | <.0001 |
| Low fear of adverse events | 3.18 | 1.841 | 5.491 | <.0001 |
| Anticipated regret | 2.588 | 1.513 | 4.426 | 0.0005 |
| Perceived behavioural control | 2.541 | 1.438 | 4.492 | 0.0013 |
| Positive attitudes toward vaccine effectiveness (ref≥20) | 2.063 | 1.108 | 3.841 | 0.0223 |
| Recruitment site (ref university hospital) | 1.961 | 1.137 | 3.385 | 0.0155 |
