# Supplementary material for: Do intentions lead to action? Results of a longitudinal study assessing determinants of Tdap vaccine uptake during pregnancy in Quebec, Canada
Source: BMC Pregnancy Childbirth. 2022 Jun 13;22:477. doi: 10.1186/s12884-022-04809-6 (PMC9189261; doi:10.1186/s12884-022-04809-6)
Supplement: Supplementary file 3 — Additional file 3: Appendix 3. Table 6. Univariate analysis: information sources about Tdap vaccination during pregnancy and vaccination status*. [file 12884_2022_4809_MOESM3_ESM.docx]

**Appendix 3**

**Table 6. Univariate analysis: information sources about Tdap vaccination during pregnancy and vaccination status***

|  | **Vaccination status** | | | | |
| --- | --- | --- | --- | --- | --- |
|  | **Vaccine** | | **No vaccine** | | **Total**  **(%)** |
|  | **N** | **%** | **n** | **%** |  |
| **Total** | **468** | **82.4** | **100** | **17.6** | **100%** |
| **Did a health care provider talk to you about the Tdap vaccine during pregnancy?** | | | | | |
| **Yes** | 447 | 95.5 | 63 | 63.0 | 89.8 |
| **No** | 15 | 3.2 | 23 | 23.0 | 6.7 |
| **You don’t know** | 6 | 1.3 | 2 | 2.0 | 1.4 |
| **Who gave you the information?**** | | | | | |
| **Nurse from a local health service centre** | 124 | 26.5 | 15 | 15.0 | 24.5 |
| **Family physician that does not follow your pregnancy** | 73 | 15.6 | 7 | 7.0 | 14.1 |
| **Health care provider that is following your pregnancy** | 400 | 85.5 | 49 | 49.0 | 79.1 |
| **Other** | 58 | 12.4 | 13 | 13.0 | 12.5 |
| **Did you receive written information about Tdap vaccination during pregnancy?** | | | | | |
| **Yes** | 429 | 91.7 | 69 | 69.0 | 87.7 |
| **No** | 21 | 4.5 | 22 | 22.0 | 7.6 |
| **You don’t know** | 17 | 3.6 | 2 | 2.0 | 3.4 |

*More than one item could be selected

**This question was answered only if participants said yes to the question “Did a health care provider talk to you about the pertussis vaccine during pregnancy? “
